# Analysis of cyclins A, B1, D1 and E in breast cancer in relation to tumour grade and other prognostic factors

**DOI:** 10.1186/1756-0500-2-140

**Published:** 2009-07-17

**Authors:** Pia Boström, Mirva Söderström, Tuire Palokangas, Tero Vahlberg, Yrjö Collan, Olli Carpen, Pirkko Hirsimäki

**Affiliations:** 1 Turku University Central Hospital, Department of Pathology, Kiinamyllynkatu 4 – 8, 20520 Turku, Finland; 2 University of Turku, Turku, Finland; 3 University of Turku, Department of Biostatistics, Lemminkäisenkatu 1, 20014 Turku, Finland; 4 Turku University Central Hospital, Department of Pathology, Biocity, Tykistökatu 6.B.6, 20520 Turku, Finland

## Abstract

**Background:**

The cell cycle is promoted by activation of cyclin dependent kinases (Cdks), which are regulated positively by cyclins and negatively by Cdk inhibitors. Proliferation of carcinoma is associated with altered regulation of the cell cycle. Little is known on the combined alterations of cyclins A, B1, D1 and E in breast cancer in relation to the tumour grade and other prognostic factors.

**Findings:**

Immunohistochemical analysis of cyclins A, B1, D1 and E, estrogen receptor, progesterone receptor, Ki-67, Her-2/neu and CK5/6 was performed on 53 breast carcinomas. mRNA levels of the cyclins were analysed of 12 samples by RT-PCR. The expression of cyclins A, B1 and E correlated with each other, while cyclin D1 correlated with none of these cyclins. Cyclins A, B1 and E showed association with tumour grade, Her-2/neu and Ki-67. Cyclin E had a negative correlation with hormone receptors and a positive correlation with triple negative carcinomas. Cyclin D1 had a positive correlation with ER, PR and non-basal breast carcinomas.

**Conclusion:**

Cyclin A, B1 and E overexpression correlates to grade, Ki-67 and Her2/neu expression. Overexpression of cyclin D1 has a positive correlation with receptor status and non-basal carcinomas suggesting that cyclin D1 expression might be a marker of good prognosis. Combined analysis of cyclins indicates that cyclin A, B and E expression is similarly regulated, while other factors regulate cyclin D1 expression. The results suggest that the combined immunoreactivity of cyclins A, B1, D and E might be a useful prognostic factor in breast cancer.

## Introduction

Breast cancer includes a heterogeneous group of tumours with variable prognosis and is a leading cause of death in women [1]. Tumour grade and size, hormone receptor status, lymph node status, and age are traditionally related to breast cancer prognosis [2]. A key event in tumorigenesis is the alteration of the genetic material, which modifies the expression of proteins in cell cycle progression [3]. The cell cycle is promoted by activation of cyclin dependent kinases, which are positively regulated by cyclins and negatively by Cdk inhibitors. This tightly controlled expression is altered in tumour cells [4]. In breast cancer, overexpression of cyclins A and E has been associated with poor prognosis [5,6] and cyclin B1 with tumour grade, Ki-67, mitoses and adverse clinical outcome [7]. The role of cyclin D1 in breast cancer remains unclear showing varying correlation to prognosis [8].

Recent gene expression studies have characterized five distinct breast carcinoma classes, two of them are ER positive (luminal A and B) and three ER negative (Her2/neu-overexpressing, normal breast-like and basal-like types) [9-11]. Basal-like cancers are positive for basal cytokeratins, but negative for hormone receptors and Her-2/neu and have been reported to be associated with worse prognosis [10]. This basal-like subgroup (ER-, PR-, Her-2/neu-, CK5/6+) includes basal cytokeratin negative tumours, which are called triple negative carcinomas (ER-, PR-, Her-2/neu-).

Although many studies have evaluated the expression and prognostic role of individual cyclins in breast cancer, little is known of their combined expression with traditional prognostic factors. Here, we have immunohistochemically evaluated cyclin A, B1, D1 and E expression in 53 breast cancers, correlated the results with grade and other prognostic factors as well as with triple negative and basal-like breast carcinomas. In addition, we analysed a subset of samples at the mRNA level to see whether the transcriptional level of cyclins correlates with the immunohistochemical results.

## Materials and methods

Patient and tissue material, immunohistochemistry, HER-2/neu chromogen in situ hybridisation, real-time quantitative polymerase chain reaction and statistical analyses are provided in additional file 1. The clinical characteristic of the patients are described in Table 1.

**Table 1:** Patients and tumour characteristics

**Variable**	**Number of patients (%)**
**Number of the patients** **Grade**	53 (aged 40–94, mean 67)
I	7 (13.2%)
II	24 (45.3%)
III	18 (34%)
in situ II	1 (1.9%)
in situ III	3 (5.7%)
**Axillary nodal status**	
N0	25 (47.2%)
N1–3	12 (22.6%)
N4–9	11 (20.8%)
>N10	3 (5.7%)
Unknown (axillary evacuation done 1993 and 1994)	2 (3.8%)
**Tumour size**	
≤ 2 cm	13 (24.5%)
> 2 cm	40 (75.5%)
**Estrogen receptor status (ER)^1)^**	
Positive	35 (66%)
Negative	14 (26.4%)
Positive in DCI	3 (5.7%)
Negative in DCIS	1 (1.9%)
**Progesterone receptor status (PR)^1)^**	
Positive	36 (68%)
Negative	13 (24.5%)
Positive in DCIS	3 (5.7%)
Negative in DCIS	1 (1.9%)
**Ki-67 status**	
<5%	7 (13.2%)
5–19%	16 (30.2%)
20–29%	8 (15.1%)
>20%	22 (41.5%)
**Histologic type**	
Ductal	37 (69.8%)
Lobular	8 (15.1%)
Subtypes	4 (7.5%)
Ductal carcinoma in situ	4 (7.5%)
**Her-2^2)^**	
IHC positive (2+ and 3+)	20 (37.7%)
IHC negative (0 and 1+)	29 (54.7%)
IHC positive in DCIS	2 (3.8%)
IHC negative in DCIS	2 (3.8%)
CISH positive	10 (18.9%)
CISH positive in DCIS	2 (3.8%)
**CK 5/6^3)^**	
Triple-negative (ER-, PR-, Her-2/neu-)	11 (20.8%)
Basal-like carcinoma (ER-, PR-, Her-2/neu-, CK5/6+)	8 (15.1%)

^1,3) ^Cut off point used for ER and PR immunohistochemistry is 10% of positively stained tumour cell nuclei and 10% cytoplasmic staining for CK5/6.

^2) ^All IHC Her 2+ and 3+ cases were retested by CISH. Scoring of HER2/neu immunohistochemistry: Score 0: no staining is observed or cell membrane staining is observed in less than 10% of tumour cells. Score 1+: a faint perceptible membrane staining can be detected in more than 10% of the tumour cells or cells are only stained in part of their membrane. Score 2+: a weak to moderate complete membrane staining is observed in more than 10% of the tumour cells. Score 3+: a strong complete membrane staining is observed in more than 10% of the tumour cells.

## Results

**Figure 1:**
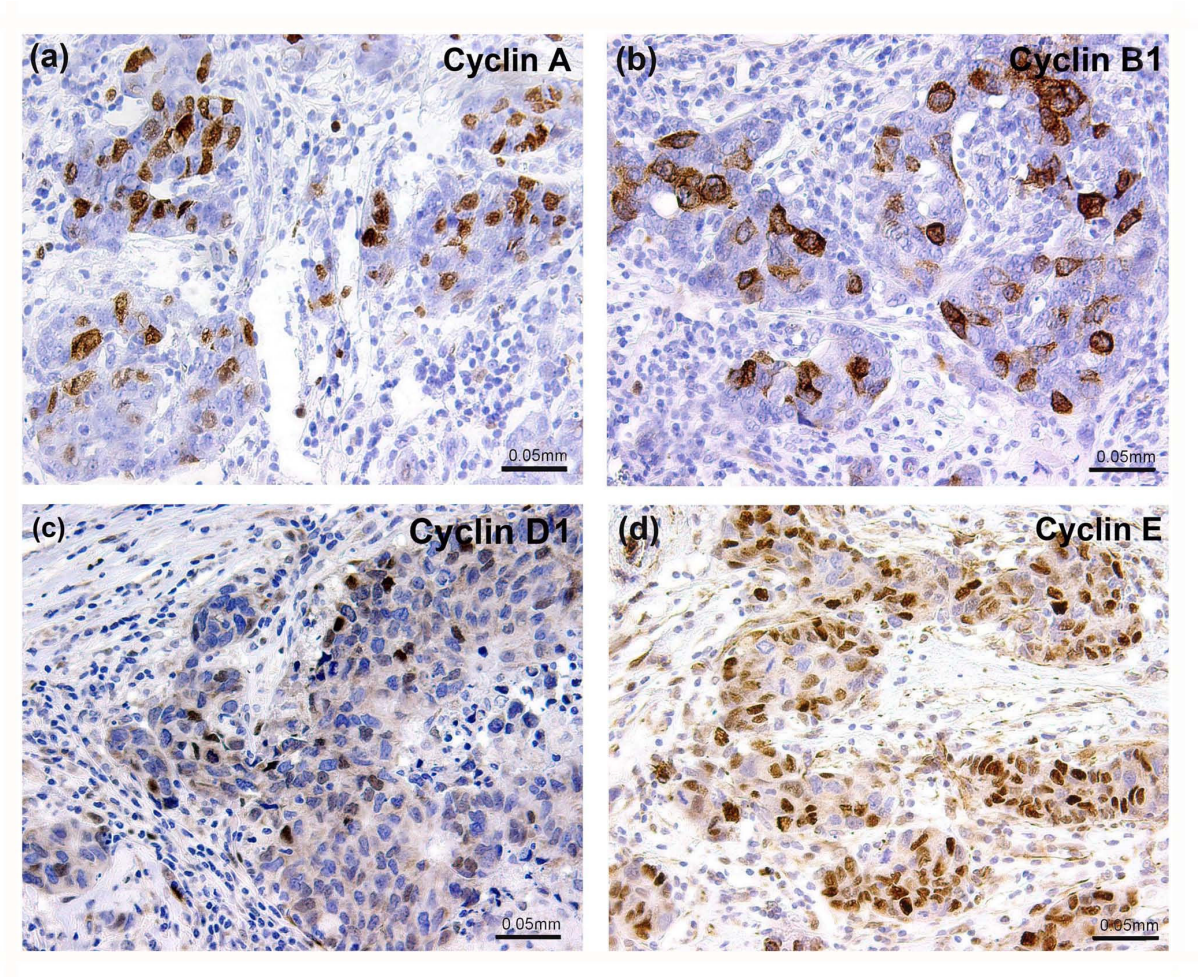
**Immunohistochemical staining of cyclins A, B1, D1 and E in infiltrating ductal carcinoma GIII**. All stainings are from the same tumour.

**Figure 2:**
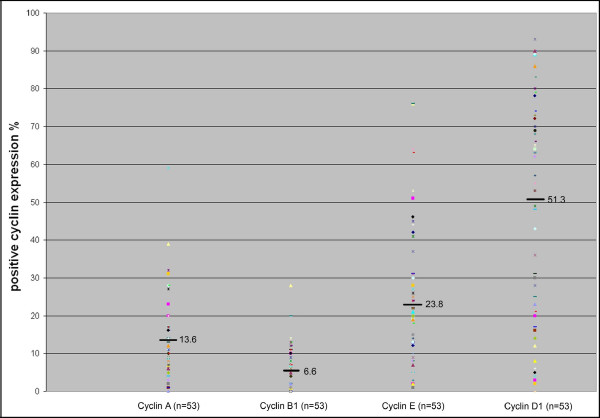
**A scattergram showing positive immunohistochemical cyclin expression (%) with mean values of cyclins A, B1, E and D1 in all tumours (n = 53)**. The different characters depict individual tumours.

The immunohistochemical staining of cyclins A, B1, D1 and E was successful in all 53 breast cancers. Representative results for each cyclin staining are presented in Figure 1. The cyclin A expression ranged from 0% to 59% in tumour samples (Figure 2). Cyclin B1 expression was low as compared to cyclin A expression and ranged from 0% to 30%. The expression of cyclin E ranged from 1% to 76% and the expression of cyclin D1 ranged between 3–90%. A significant positive correlation was seen between grade and Ki-67 (r = 0.62, p < 0.0001), and Her-2/neu immunohistochemistry (r = 0.35, p = 0.0104).

**Figure 3:**
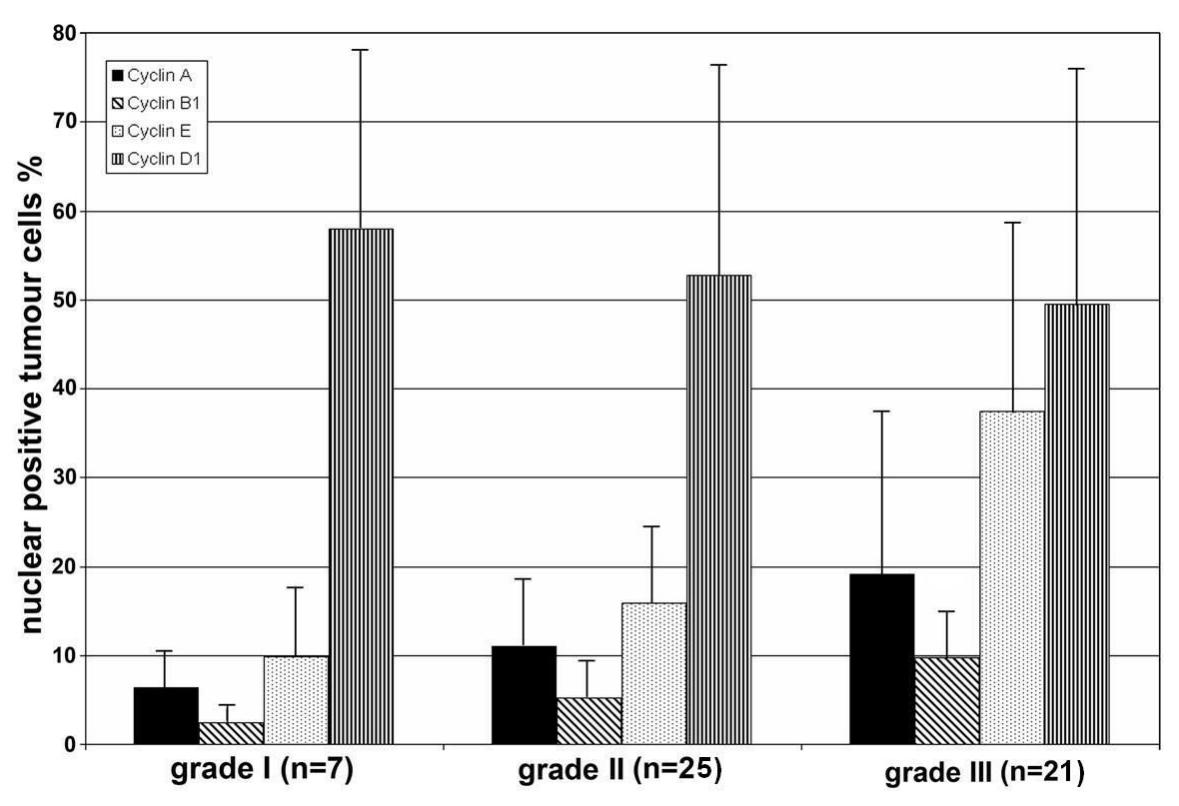
**The association of histological grade of the breast cancer with the immunohistochemical expression of cyclin A, B1, E and D1**. P-values for cyclin A: overall p = 0.0025, grade I vs II p = 0.3774, grade I vs III p = 0.0069, grade II vs III p = 0.0321. P-values for cyclin B1: overall p = 0.0058, grade I vs II p = 0.5466, grade I vs III p = 0.015, gradus II vs III p = 0.048. P-values for cyclin E: overall p < 0.0001, grade I vs II p = 0.294, grade I vs III p = 0.0063, grade II vs III p < 0.0003. Cyclin D1 did not show any correlation with the grades.

The immunohistochemically detected expression of cyclin A (p = 0.0011), B1 (p = 0.0047) and E (p = 0.0005) showed significant associations with tumour grade (Figure 3). In addition, cyclin A, B1 and E expression showed positive correlation with Ki-67 expression (r = 0.71, p < 0.0001 for cyclin A, r = 0.57, p < 0.0001 for cyclin B1 and r = 0.60, p < 0.0001 for cyclin E), while cyclin D1 staining showed no significant correlation with grade or Ki-67 expression. None of the cyclins showed correlation with tumour size or metastases.

Cyclin A (r = 0.32, p = 0.0264), cyclin B1 (r = 0.43, p = 0.0026) and cyclin E staining (r = 0.34, p = 0.0199) had a significant positive correlation with Her-2/neu expression, while cyclin D1 staining did not have any correlation with Her-2/neu expression. The tumour grade had also a significant positive correlation with Her-2/neu expression (r = 0.46, p = 0.0011). Cyclin D1 staining had a significant positive correlation with expression of ER (r = 0.37, p = 0.0088) and PR (r = 0.33, p = 0.0233) whereas cyclin E staining had a significant negative correlation with ER (r = -0.37, p = 0.0100) and PR status (r = -0.35, p = 0.0153). Cyclins A and B1 did not show any correlations with ER or PR status.

Cyclin A, B1 and E expression correlated with each other, but not with cyclin D1. Cyclin A had a significant positive correlation with cyclin E (r = 0.49, p = 0.0004) and especially with cyclin B1 (r = 0.60, p < 0.0001). On the other hand, cyclin B1 had a significant positive correlation with cyclin E (r = 0.52, p = 0.0001). High cyclin E expression correlated with triple negative cancers (p = 0.0474), while cyclin D1 expression correlated with non-triple negative carcinomas (p = 0.0156) and non-basal-like carcinomas (p = 0.0279). Cyclins A and B1 didn't show any correlation with triple negative or basal-like breast carcinomas. Nor showed cyclin E any correlation with basal-like carcinomas.

**Figure 4:**
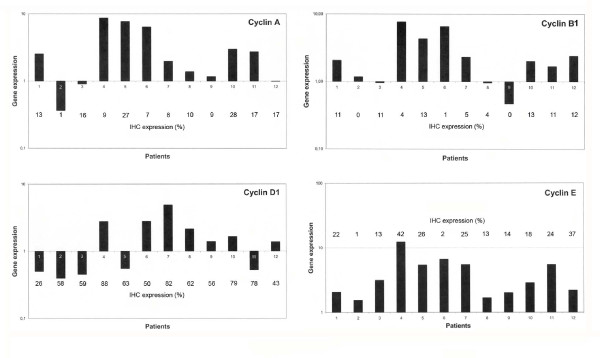
**Gene expression levels of different cyclins in 12 patients analysed by RT-PCR**. The horizontal line 1 represents the expression of normal breast tissue. Under the columns are the numbers of the patients and the immunohistochemical staining results (%) of each cyclin. Patients: no 1–2 = DC GI, no 3 = DC GII, no 4–6 DC GIII, no 7–8 = LC GI, no 9 = LC GII and no 10–12 = DCIS GIII.

The mRNA expression of the four cyclins was analyzed by RT-PCR in 12 breast cancer samples (Figure 4). Cyclin A gene expression was increased in 9/12 tumours, cyclin B1 in 9/12 tumours, cyclin D1 in 7/12 tumours and cyclin E in all 12 tumours. Although differences were seen between the gene expressions, a number of similarities were also observed, for example higher expression of cyclins A, B1 and E in ductal carcinoma grade III tumours. Also, two ductal carcinomas in situ GIII had higher cyclin A gene expression than other breast tumours (however, third DCIS GIII had lower gene expression than normal breast tissue). Ductal carcinoma in situ GIII patients had also a moderate gene expression for cyclin B1 and E. In our material, cyclin D1 gene expression was variable. Comparisons between the immunohistochemical expression of different cyclins and quantitative PCR demonstrated disagreement in certain tumours. Further assessment of the statistical correlations between the gene expression and immunohistochemical expression were not possible due to the relatively limited number of samples.

## Discussion

To our knowledge this is the first study, where the expression of cyclins A, B1, D1 and E is collectively analysed in breast cancer to determine the correlation with tumour grade and other traditional prognostic factors as well as with basal-like (ER-, PR- and Her-2/neu-, CK5/6+) and triple negative breast carcinomas (ER-, PR- and Her-2/neu-, CK5/6-). Overexpression of cyclins A, B1, and E has been reported to associate with poor prognosis in breast cancer [5-7]. Therefore, it was not surprising that the immunoreactivity of cyclins A, B1 and E showed significant association with tumour grade, Her-2/neu and Ki-67 expression. Immunohistochemically detected expression of cyclins A, B1 and E correlated with each other, but not with cyclin D1. Furthermore, cyclin E had a negative correlation with hormone receptor status and a positive correlation with triple negative breast carcinomas associating cyclin E expression with unfavourable prognosis. The role of cyclin D1 as a marker of breast cancer prognosis is somewhat speculative and contradictory results have been published [8]. Nevertheless, we observed a significant positive correlation between immunoreactivity of cyclin D1 and hormone receptors as well as with non-basal-like carcinomas and non-triple negative carcinomas. In addition, cyclin D1 did not correlate with grade, Ki-67, Her-2/neu suggesting that increased cyclin D1 level might be a marker of good outcome.

In breast cancer, high cyclin A expression has been demonstrated to correlate with tumour grade, Ki-67 and Her-2/neu [5]. Our findings are in agreement with these observations. On the other hand, although cyclin A correlated with poor prognosis, it was unexpected to find that neither cyclin A, nor any of the other cyclins studied showed correlation with tumour metastasis. There are, however, also opposite results concerning the roles of cyclin A as a prognostic marker. Kuhling and co-workers (2003) reported that cyclin A did not achieve statistical significance in predicting disease-specific and metastasis-free survival [12]. These contradicting results may be, at least partly, explained by the fact that tumours with high proliferation rate and cyclin A expression may have a more favourable response to chemotherapy [13].

We analysed a subset of samples to study whether the transcription of cyclin mRNAs correlate with the cyclin protein levels using quantitative PCR, which is a precise method to determine the amount of mRNA. Our results demonstrated increased transcription of cyclins in most of the tumour samples analysed. When the mRNA levels were compared with the protein levels, many similarities were observed, although some differences also were evident. An example of this is cyclin B1, where the gene expression was up-regulated in 9/12 cases (75%) and the mRNA levels tended to be high in grade III tumours. Furthermore, immunohistochemically detected expression of cyclin B1 showed a significant association with tumour grade, Ki-67 and Her-2/neu as well as with cyclin E expression. Others have also found an association between cyclin B1 overexpression and poorer survival in breast cancer [14].

Despite several studies, the exact role of cyclin D1 in breast cancer remains unclear. The expression of cyclin D1 has previously been shown to positively correlate with ER status and negatively with tumour grade and size, thus suggesting that cyclin D1 overexpression is a marker of good outcome particularly when ER is coexpressed [15]. Our findings in the present study go along with these observations. There are also reports showing that overexpression of cyclin D1 predicts tamoxifen treatment resistance in breast cancer patients [16-19] and associates with poor prognostic features in estrogen receptor positive breast cancer [8]. Furthermore, cyclin D1 is thought to mediate cell proliferation by different mechanisms in estrogen receptor positive and negative breast cancer [8]. In our RT-PCR study, cyclin D1 gene was up-regulated in 7/12 (58%) tumours studied, which is higher than previously reported (5–20%) [20]. When cyclin D1 mRNA level was compared with the immunohistochemically detected expression, cyclin D1 immunoreactivity was constantly higher suggesting that translation of cyclin D1 is not always secondary to gene transcription and other mechanisms as defects in the proteasomal degradation of cyclin D1 [21] may affect the maintenance of cyclin D1 overexpression.

Several studies have shown that cyclin E immunoreactivity correlates with negative ER status, higher tumour grade, HER-2/neu and higher proliferation index [22]. Our findings in the present study are in agreement with these observations, as immunohistochemically detected expression of cyclin E showed significant association with histological grade, Ki-67, Her-2/neu staining and a negative correlation with ER and PR status. We also observed a positive correlation between cyclin E immunoreactivity and triple negative carcinomas. Furthermore, compared to normal breast tissue, cyclin E mRNA expression was up-regulated in all 12 samples analysed by quantitative PCR. Previous studies have demonstrated some differences between quantitative PCR results and immunohistochemically detected expression of cyclin E [23]. This discrepancy may be explained by the fact that mRNA used for PCR originated from cancer cells as well as from tumour stromal cells, while in immunohistochemical staining only cancer cells are scored [23]. However, in our study cyclin E immunoreactivity seemed to reflect the amount of cyclin E mRNA detected by quantitative PCR, as the highest mRNA levels were detected in grade III samples.

## Conclusion

This study shows that the aggressive breast cancers overexpress cyclins A, B1 and E whereas cyclin D1 expression is independent of the other cyclins. The overexpression of cyclin D1 has a significant positive correlation with hormone receptor status, non-triple negative and non-basal-like breast carcinomas suggesting that cyclin D1 expression might be a marker of good prognosis. Despite of the small number and heterogeneity of the our patient material it may be beneficial to study the expression of cyclins A, B1, D1 and E together with other cell cycle regulators, when determining breast cancer prognosis.

## Competing interests

The authors declare that they have no competing interests.

## Authors' contributions

PB participated in the design of the study, carried out the screening of the tumour material and the evaluation of the immunohistochemical staining and drafted the manuscript. MS participated in study design, provided RNA from the breast tumours and helped to draft the manuscript. TP performed the statistical analysis. TV coordinated the statistical analysis and helped to draft the manuscript. YC participated in the design of the study and helped to draft the manuscript. OC helped to draft the manuscript. PH participated in the design of the study, carried out the evaluation and validation of the immunohistochemical staining and helped to draft the manuscript. All authors have read and approved the final manuscript.

## Supplementary Material

Additional File 1 **Detailed Material and Methods**. A detailed description of the patient and tissue materials used, including immunohistochemical stainings, HER-2/neu chromogen in situ hybridisation, real-time quantitative polymerase chain reaction and statistical analyses. Click here for file

## References

[B1] Finnish Cancer Registry (2007). Cancer in Finland 2004 and 2005. Cancer Statistics of the National Research and Development Centre for Welfare and Health. Cancer Society of Finland Publication No 72 Helsinki.

[B2] Finek J, Holubec L, Topolcan O, Elgrova L, Skalova A, Pecen L (2007). The importance of prognostic factors in premenopausal women with breast cancer. Anticancer Res.

[B3] Peters MG, Vidal Mdel C, Gimenez L, Mauro L, Armanasco E, Cresta C, Bal de Kier Joffe E, Puricelli L (2004). Prognostic value of cell cycle regulator molecules in surgically resected stage I and II breast cancer. Oncol Rep.

[B4] Malumbres M, Barbacid M (2009). Cell cycle, CDKs and cancer: a changing paradigm. Nature Reviews Cancer.

[B5] Poikonen P, Sjostrom J, Amini RM, Villman K, Ahlgren J, Blomqvist C (2005). Cyclin A as a marker for prognosis and chemotherapy response in advanced breast cancer. Br J Cancer.

[B6] Keyomarsi K, Tucker S, Buchholz T, Callister M, Ding Y, Hortobagyi G, Bedrosian I, Knickerbocker C, Toyofuku W, Lowe M, Herliczek T, Bacus S (2002). Cyclin E and survival in patients with breast cancer. N Engl J Med.

[B7] Suzuki T, Urano T, Miki Y, Moriya T, Akahira J, Ishida T, Horie K, Inoue S, Sasano H (2007). Nuclear cyclin B1 in human breast carcinoma as a potent prognostic factor. Cancer Sci.

[B8] Aaltonen K, Amini R, Landberg G, Eerola H, Aittomäki K, Heikkilä P, Nevanlinna H, Blomqvist C (2008). Cyclin D1 expression is associated with poor prognostic features in estrogen receptor positive breast cancer. Breast Cancer Res Treat.

[B9] Reis-Filho JS, Tutt ANJ (2008). Triple negative tumours: a critical review. Histopathology.

[B10] Fadare O, Tavassoli F (2008). Clinical and pathologic aspects of basal-like breast cancers. Nat Clin Pract Oncol.

[B11] Morris SR, Carey LA (2007). Molecular profiling in breast cancer. Rev Endocr Metab Disord.

[B12] Kuhling H, Alm P, Olsson H, Ferno M, Baldetorp B, Parwaresch R, Rudolph P (2003). Expression of cyclins E, A and B, and prognosis in lymph node-negative breast cancer. J Pathol.

[B13] Ahlin C, Aaltonen K, Amini R-M, Nevanlinna H, Fjällskog M-L, Blomqvist C (2007). Ki67 and cyclin A as prognostic factors in early breast cancer. What are the optimal cut-off values?. Histopathology.

[B14] Aaltonen K, Amini R-M, Heikkilä PK, Aittomäki K, Tamminen AH, Nevanlinna H, Blomqvist C (2009). High cyclin B1 expression is associated with poor survival in breast cancer. British Journal of Cancer.

[B15] Hwang TS, Han HS, Hong YC, Lee HJ, Paik NS (2003). Prognostic value of combined analysis of cyclin D1 and estrogen receptor status in breast cancer patients. Pathol Int.

[B16] Ahnström M, Nordenskjöld B, Rutqvist LE, Skoog L, Stål O (2005). Role of cyclin D1 in ErbB2-positive breast cancer and tamoxifen resistance. Breast Cancer Res Treat.

[B17] Stendahl M, Kronblad A, Ryden L, Emdin S, Bengtsson NO, Landberg G (2004). Cyclin D1 overexpression is a negative predictive factor for tamoxifen response in postmenopausal breast cancer patients. Br J Cancer.

[B18] Ishii Y, Waxman S, Germain D (2008). Tamoxifen stimulates the growth of cyclin D1-overexpressing breast cancer cells by promoting the activation of signal transducer and activator of transcription 3. Cancer Res.

[B19] Jirström K, Stendahl M, Ryden L, Kronblad A, Bendahl PO, Stål O, Landberg G (2005). Adverse effect of adjuvant tamoxifen in premenopausal breast cancer with cyclin D1 gene amplification. Cancer Res.

[B20] Roy PG, Thompson AM (2006). Cyclin D1 and breast cancer. The Breast.

[B21] Barbash O, Diehl JA (2008). SCF(Fbx4/alphaB-crystallin) E3 ligase: when one is not enough. Cell Cycle.

[B22] Potemski P, Kusinska R, Watala C, Pluciennik E, Bednarek AK, Kordek R (2006). Cyclin E expression in breast cancer correlates with negative steroid receptor status, HER2 expression, tumor grade and proliferation. J Exp Clin Cancer Res.

[B23] Potemski P, Pluciennik E, Bednarek AK, Kusinska R, Jesionek-Kupnicka D, Pasz-Walczak G, Watala C, Kordek R (2006). Cyclin E expression in operable breast cancer quantified using real-time RT-PCR: a comparative study with immunostaining. Jpn J Clin Oncol.

