# Supplementary material for: Analysis of cyclins A, B1, D1 and E in breast cancer in relation to tumour grade and other prognostic factors
Source: BMC Res Notes. 2009 Jul 17;2:140. doi: 10.1186/1756-0500-2-140 (PMC2716358; doi:10.1186/1756-0500-2-140)
Supplement: Additional File 1 — Detailed Material and Methods. A detailed description of the patient and tissue materials used, including immunohistochemical stainings, HER-2/neu chromogen in situ hybridisation, real-time quantitative polymerase chain reaction and statistical analyses. [file 1756-0500-2-140-S1.doc]

**Material and Methods**

***Patients and tissue material***

The material consisted of 53 breast cancer patients who were operated and treated at Turku University Central Hospital during the years 2004-2008. Each specimen was used with the approval of the Ethics committee of Turku University (no 241/2005). Since fresh tumour tissue was collected for analysis, patients undergoing radical mastectomy and having over 10 mm tumour were included. Lymph node dissection was performed in 24 patients because of metastasis in the sentinel node or preoperatively known metastasis in axillary lymph node. Three of the specimens were from re-operations performed due to the breast cancer recurrence. One patient had received preoperative neoadjuvant chemotherapy because of inflammatory breast cancer. The age of the patients ranged from 40 to 94 years (mean 67 years) at the time of diagnosis.

Samples were excised by one of the authors (PB) from the invasive border of the tumour within 20 minutes after the surgical removal of the breast. The specimen was cut in two pieces. One half was frozen in liquid nitrogen and stored at -72^◦^C for further mRNA analysis, while the other half was fixed in 10 % phosphate buffered formaldehyde and embedded in paraffin. In all cases, 4 µm thick serial paraffin sections were cut from tumour tissue and stained with haematoxylin and eosin (HE). The tumour histology was assessed according to the WHO classification. Tumour grading (I-III) was based on the recommendations made by Elston and Ellis 1991 [1]. Tumour size and axillary lymph node status were analysed.

***Immunohistochemistry***

Immunohistochemical stainings of cyclins A, B1, D1 and E, estrogen (ER) and progesterone (PR) receptors, Ki-67, CK5/6 and HER-2/neu were performed from subsequent sections with TechMate 500+ immunostainer using monoclonal antibodies and a peroxidase/diaminobenzidine LSAB+ detection kit (DAKO, K5001). The used antibodies, dilutions and pre-treatments are presented in Table 1. Areas showing necrosis or inflammation were excluded from the analysis. The areas of the proliferating cells at the border of the most cellular part of the tumour were identified in each immunohistochemical staining. For Ki-67, hormone receptors and cyclin analyses, the numbers of immunoreactive nuclei (%) of 100 malignant cells from three separate cell rich areas were calculated with a 40 x objective independently by two persons without any knowledge of the clinical data. In cases with discrepancies, a consensus was achieved after re-examination. Her-2/neu expression was evaluated as membrane staining of invasive tumour cells and scored to four classes (0/1+/2+/3+) [2]. Carcinomas were divided into luminal A and B, Her-2/neu overexpressing, normal breast-like and triple negative carcinomas. The triple negative tumours were analysed immunohistochemically for CK5/6 expression and specimens where at least 10% of the tumour cells showed cytoplasmic staining were interpreted as positive and classified as basal-like carcinomas (more than 10% of cells are CK5/6 positive and lack ER, PR and HER-2/neu expression) [3].

***HER-2/neu chromogen in situ hybridisation***

Chromogen in situ hybridization of breast cancer samples was performed in order to confirm the immunohistochemical 2+ - 3+ cases of HER-2/neu gene expression as well as also ambiguous results. The test was performed on 4 μm thick paraffin sections using the SPoT-Light HER-2 CISH- kit in which the gene specific HER-2/neu probe has been labelled with digoxigenin (Zymed). The target gene was localized with a HRP/DAB-detection system. The gene expression of Her-2/neu was interpreted as positive if 6 or more gene copies and/or clusters were observed at least in 10% of the tumour cells.

***Real-time quantitative polymerase chain reaction***

Cyclins A, B1, D1 and E m-RNA levels were analysed in 12 breast cancer samples (one ductal carcinoma grade II and lobular carcinoma grade II, two ductal carcinomas grade I and lobular carcinomas grade I, three ductal carcinomas grade III and ductal carcinoma in situ grade III) using normal human breast tissue as a reference. RNA was isolated using the RNeasy kit (Qiagen). For each sample, 1µg of RNA was treated with RQ1 DNase (Promega) and reverse transcribed using Moloney murine leukaemia virus RNase H (Promega). Primers and probes for the cyclin genes were chosen using the ProbeFinder software program (Roche Applied Science) and for the *GAPDH (*glyceraldehyde 3-phosphate dehydrogenase*)* the Primer Express 2.0 software program (Applied Biosystems). Sequences of primers and probes used are presented in Table 2. ABsolute QPCR ROX Mix (ABgene) was used to prepare the reaction mixes, and the PCR was performed with ABI Prism 7700 Sequence Detection system (Applied Biosystems). Relative quantities of cyclin mRNAs were normalized against *GAPDH* and relative gene expression was calculated using the 2^–δδct^ method with cDNA from normal breast tissue as a reference [4].

***Statistical analyses***

The associations between grade and the immunohistochemical expression of cyclins were tested with Kruskal-Wallis and pairwise comparisons with Mann-Whitney U–tests. In pairwise comparisons Bonferroni-adjustment was applied. Correlations were calculated using Spearman rank-order correlation coefficients. Non-parametric tests were applied because of non-normal distribution of variables. The statistical analyses were carried out using SAS/STAT (r) software, Version 9.1.3 SP4 of the SAS System for Windows (SAS Institute Inc., Cary, NC). P-values less than 0.05 were considered statistically significant.

**Tables and captions**

| **Table 1.** *Immunostaining protocol.* Immunohistochemical stainings of cyclins A, B1, D1 and E as well as ER, PR, Ki-67, CK5/6 and HER-2/neu were performed from formalin fixed, paraffin embedded specimens with TechMate 500+ immunostainer using monoclonal antibodies and a peroxidase/diaminobenzidine LSAB+ detection kit (DAKO, K5001).  **Antibody Source Dilution Pre-treatment in microwave oven, 2 x 7 min, 850W**  Estrogen DAKO M7047 1:40 10 mM TRIS-HCl 1mM EDTA (pH 9)  Progesterone Novocastra NCL-PGR 1:20 10 mM TRIS-HCl 1mM EDTA (pH 9)  Ki-67 DAKO M7240 1:100 10 mM sodium citrate buffer (pH 6)  Her2/neu Novocastra NCL-CB11 1:50 10 mM sodium citrate buffer (pH 6)  Cyclin A Novocastra NCL-CYCLIN A 1:60 10 mM sodium citrate buffer (pH 6)  Cyclin B1 Novocastra NCL-CYCLIN B1 1:10 10 mM sodium citrate buffer (pH 6)  Cyclin D1 LabVision RM-9104-S 1:50 10 mM TRIS-HCl 1mM EDTA (pH 9)  Cyclin E Zymed HE12 1:50 10 mM TRIS-HCl 1mM EDTA (pH 9)  Cytokeratin 5/6 DAKO M7237 1:50 10 mM TRIS-HCl 1mM EDTA (pH9)  **Table 2.** *Primer and probe sequences used in real time quantitative polymerase chain reaction.*  **Gene Primer sequence (5´-3´) Hybridisation probe sequence (5´-3´)**  ***Cyclin A***  Sense CCATACCTCAAGTATTTGCCATC AAAAGCCAGGGCATCTTCACGCTCTATT  Antisense TCCAGTCTTTCGTATTAATGATTCAG  ***Cyclin B1***  Sense CATGGTGCACTTTCCTCCTT CCCGGACTGAGGCCAAGAACAGC  Antisense AGGTAATGTTGTAGAGTTGGTGTCC  ***Cyclin D1***  Sense TCCAGAGTGATCAAGTGTGACC CCTCCAGCATCCAGGTGGCGA  Antisense TGGGGTCCATGTTCTGCT  ***Cyclin E***  Sense ACAGCTTGGATTTGCTGGA TCAGTGCCGACTCTGCCACATGG  Antisense TCTGCTTCTTACCGCTCTGTG  ***GAPDH***  Sense ACCCACTCCTCCACCTTTGA ACGACCACTTTGTCAAGCTCATTTCCT  Antisense TTGCTGTAGCCAAATTCGTTGT |
| --- |

**References (Methods)**

1. Elston CW, Ellis IO: **Pathological prognostic factors in breast cancer. I. The value of histological grade in breast cancer: experience from a large study with long-term follow up.** *Histopathology* 1991, **19**:403-410.
2. Isola J, Tanner M, Forsyth A, Cooke TG, Watters AD, Bartlett JM: **Interlaboratory comparison of HER-2 oncogene amplification detected by chromogenic and fluorescence in situ hybridization.** *Clin Cancer Res* 2004, **10**:4793-4798.
3. Rakha EA, Putti TC, Abd El-Rehim DM, Paish C, Green AR, Powe DG, Lee AH, Robertson JF and Ellis IO: **Morphological and immunophenotypic analysis of breast carcinomas with basal and myoepithelial differentiation.** J Pathol 2006; **208**:495-506.
4. Livak KJ, Schmittgen TD: **Analysis of relative gene expression data using real-time quantitative PCR and the 2 ^-ΔΔCT^ Method.**  *Methods* 2001, **25**:402-408.
